# Biosynthesis of UDP-GlcNAc, UndPP-GlcNAc and UDP-GlcNAcA Involves Three Easily Distinguished 4-Epimerase Enzymes, Gne, Gnu and GnaB

**DOI:** 10.1371/journal.pone.0067646

**Published:** 2013-06-14

**Authors:** Monica M. Cunneen, Bin Liu, Lei Wang, Peter R. Reeves

**Affiliations:** 1 School of Molecular and Microbial Bioscience, University of Sydney, Sydney, Australia; 2 TEDA School of Biological Sciences and Biotechnology, Nankai University, TEDA, Tianjin, China; 3 Tianjin Research Center for Functional Genomics and Biochip, TEDA, Tianjin, China; 4 Tianjin Key Laboratory of Microbial Functional Genomics, Nankai University, Tianjin, China; University of Helsinki, Finland

## Abstract

We have undertaken an extensive survey of a group of epimerases originally named Gne, that were thought to be responsible for inter-conversion of UDP-N-acetylglucosamine (UDP-GlcNAc) and UDP-N-acetylgalactosamine (UDP-GalNAc). The analysis builds on recent work clarifying the specificity of some of these epimerases. We find three well defined clades responsible for inter-conversion of the gluco- and galacto-configuration at C4 of different N-acetylhexosamines. Their major biological roles are the formation of UDP-GalNAc, UDP-N-acetylgalactosaminuronic acid (UDP-GalNAcA) and undecaprenyl pyrophosphate-N-acetylgalactosamine (UndPP-GalNAc) from the corresponding glucose forms. We propose that the clade of UDP-GlcNAcA epimerase genes be named *gnaB* and the clade of UndPP-GlcNAc epimerase genes be named *gnu*, while the UDP-GlcNAc epimerase genes retain the name *gne*. The Gne epimerases, as now defined after exclusion of those to be named GnaB or Gnu, are in the same clade as the GalE 4-epimerases for inter-conversion of UDP-glucose (UDP-Glc) and UDP-galactose (UDP-Gal). This work brings clarity to an area that had become quite confusing. The identification of distinct enzymes for epimerisation of UDP-GlcNAc, UDP-GlcNAcA and UndPP-GlcNAc will greatly facilitate allocation of gene function in polysaccharide gene clusters, including those found in bacterial genome sequences. A table of the accession numbers for the 295 proteins used in the analysis is provided to enable the major tree to be regenerated with the inclusion of additional proteins of interest. This and other suggestions for annotation of 4-epimerase genes will facilitate annotation.

## Introduction

The O-specific polysaccharide (OPS) or O antigen forms a major part of the lipopolysaccharide (LPS) of the Gram-negative bacterial cell wall, and generally consists of a polysaccharide chain of identical repeat units, each with about 2-6 sugar residues. The OPS is synthesised separately from the lipid A/ core, and in those systems that have been studied is usually synthesised by the Wzx/Wzy pathway in which the repeat unit is built on the lipid carrier undecaprenol pyrophosphate (UndPP) [1].

There is enormous diversity of sugars in OPS, with 71 listed in a recent review [2]. The genes responsible for synthesis of the diverse OPS within a species are generally in a gene cluster at a conserved locus, which for *E. coli* is between the *galF* and *gnd* genes and for *Yersinia* sp between the *gsk* and *hemH* genes. Repeat-unit biosynthesis starts with addition of a sugar phosphate to undecaprenol phosphate (UndP) by an initial sugar phosphate transferase (IT) to generate a membrane-associated UndPP-linked sugar. The other sugars are added by glycosyl transferases (GT) to generate an UndPP-linked repeat unit, that is translocated across the membrane to place the repeat unit on the periplasmic face, where it is polymerised by the OPS polymerase, Wzy, to form complete OPS still linked to UndPP. After polymerisation each repeat unit except the first is linked to a specific sugar in the previous repeat unit of the chain. Thus the choice of initial sugar is determined by the IT, but its linkage in the polymer is determined by the specificity of the very variable Wzy OPS polymerase.

In this paper we study a group of N-acetylhexosamine-4-epimerases and hexose-4-epimerases C-4 epimerises that are responsible for an important step in synthesis of several sugars, some very common. It has recently become clear that there is considerable confusion on their specificity. We will look first at those examples that have been studied and have known functions, and then at how we can use this information to identify clades that allow reasonable predictions of function.

### *GalE and Gne are UDP-Glc and UDP-GlcNAc 4-epimerases*

The human UDP-Glc 4-epimerase can also use UDP-GlcNAc as a substrate, but this is not the case for the *E. coli* GalE enzyme [3]. However Bengoechea et al. [4] showed that a galE-like gene in the *Yersinia enterocolitica* O:8 OPS gene cluster coded for a UDP-GlcNAc 4-epimerase that could also use UDP-Glc as substrate. This is in addition to the *galE* gene in the *gal* operon. The new gene was named *gne*, a name first used for a UDP-GlcNAc epimerase gene in *Bacillus subtilis* [5].

Bengoechea et al. [4] also showed that for both the *Y. enterocolitica* Gne and the *E. coli* GalE epimerases, the ability to handle UDP-GlcNAc is determined by the size of the active site. Amino-acid substitutions that reduce its size in the *Y. enterocolitica* O8 Gne protein prevented use of UDP-GlcNAc, and substitutions in GalE of *E. coli* that expand its size allowed UDP-GlcNAc to become a substrate. Clearly in both cases we have what are effectively a Gne epimerase and a GalE epimerase that differ in a single amino acid residue.

### *Initial transferases in the Enterobacteriaceae*

The *Enterobacteriaceae* are unusual in that most OPS gene clusters lack an initial transferase (IT) gene. The Enterobacterial Common Antigen (ECA), which is generally present in this family, has GlcNAc as its first sugar, and it has become clear that WecA, the IT for ECA, also acts as the IT for most OPS in the family. The role of WecA in initiation of OPS synthesis by formation of UndPP-GlcNAc was shown directly for several *E. coli* serogroups [6]. The extrapolation to most Enterobacterial OPS is based on the observation that those Enterobacterial OPS that lack an IT gene in their gene cluster have either GlcNAc, GalNAc or both in their structure. Where GlcNAc is first sugar, the role of WecA is assumed to be the same as for those studied by Alexander et al [6]. When, in the absence of a GlcNAc residue, GalNAc was implicated as first sugar, a galE-like gene was often observed in the gene cluster. This was originally assumed to be a *gne* gene, with the Gne product converting UDP-GlcNAc to UDP-GalNAc to provide an alternative substrate for WecA, while WecA was assumed to be able to use GalNAc as well as GlcNAc as the initial sugar. However two recent publications revealed fatal flaws in this hypothesis.

### *GlcNAc epimerase function in E. coli O157*

The *E. coli* O157 repeat unit contains a single GalNAc but no GlcNAc residue and the gene cluster has no IT gene [7], so OPS synthesis was expected to be initiated by WecA acting on UDP-GalNAc. A *galE* homologue was observed just upstream of the OPS gene cluster and this was assigned as the then predicted *gne* gene [7], although the level of similarity to other *gne* genes was low. In 2010 Rush et al. [8] showed that the O157 epimerase had no activity on UDP-GlcNAc, but instead acted on UndPP-GlcNAc to give UndPP-GalNAc. The pathway was shown to be formation of UndPP-GlcNAc by WecA, followed by conversion to UndPP-GalNAc by the “Gne” epimerase previously assumed to convert UDP-GlcNAc to UDP-GalNAc. Clearly some of the enzymes presumed to be UDP-GlcNAc epimerases (Gne) have a different function.

Rush et al. [8] also compared the O157 epimerase with epimerases from other gene clusters for GalNAc-containing OPS, and showed that those where GalNAc was the first sugar were in a different homology group than those where GalNAc was not the first sugar.

### *E. coli O86 and Yersinia pseudotuberculosis O6 and O7 have separate UDP-GlcNAc and UndPP-GlcNAc epimerases for OPS synthesis*

The second observation was with the well-studied *E. coli* O86 OPS. The gene cluster region contains two *gne-*like genes, referred to as *gne1* and *gne2*, and knockouts of either resulted in an altered LPS phenotype, indicating that both are required for normal OPS synthesis [9], and hence must have different functions. This could not be understood at the time but with clarification of the situation in *E. coli* O157:H7 as discussed above, the explanation became clear. There are two GalNAc residues that are the first and second sugars in the biological repeat unit [10]. The *gne1* gene is upstream of the repeat-unit gene cluster, at the same locus as the *E. coli* O157 *gne* gene, and the encoded proteins are identical. Gne1 of O86 must also be an UndPP-GlcNAc epimerase, converting the first sugar to UndPP-GalNAc. The *gne2* gene is the first gene within the repeat-unit gene cluster (after the flanking gene *galF*), and Gne2 must of necessity be the UDP-GlcNAc epimerase producing the UDP-GalNAc substrate required as donor of the second GalNAc residue of the repeat unit, because it is required for OPS expression and it is the only conceivable function.

The *Y. pseudotuberculosis* O6 and O7 OPS also have two GalNAc residues, and share a pair of *gne*-like genes. In this case both genes are within the main gene clusters and are separated by a shared gene proposed to encode the GT linking the two GalNAc residues, which are the first and second sugars of the O unit. The genes are named *gne1* and *gne2* in map order, and on the basis of their sequence similarities, and knowing the role of the *E. coli* O157 gene, were proposed to encode UndPP-GlcNAc and UDP-GlcNAc epimerases respectively [11]. The three genes, present at the 3’ end in both gene clusters, constitute a module for assembly of the GalNAc-(1–3)-GalNAc-UndPP structure, starting with UndPP-GlcNAc formed by WecA [11].

### *Epimerisation in the formation of UDP-GalNAcA*

There has also been a recent clarification on function of a “*gne*” gene in the UDP-GalNAcA pathway. Synthesis of UDP-GalNAcA from UDP-GlcNAc requires 2 steps, epimerisation at C4 and oxygenation at C6. In the case of the *Pseudomonas aeruginosa* O6 the two proteins are encoded in the OPS gene cluster and have been well studied. The pathway has recently been shown to be oxygenation of UDP-GlcNAc to UDP-GlcNAcA (WbpO) followed by epimerisation to UDP-GalNAcA (WbpP) [12]. This is the reverse of the originally proposed order [13]. WbpP, the epimerase, had previously been shown *in vitro* to convert UDP-GlcNAc to UDP-GalNAc as for the original Gne proteins, and WbpO to convert UDP-GalNAc to UDP-GalNAcA [13]. However it is now clear that while it is possible *in vitro* to carry out the oxygenation step before epimerisation [13,14], *in vivo* the epimerisation reaction is carried out on UDP-GlcNAcA after the oxygenation step [12,15].

### *Subdivision of Gne-like epimerases*

In 2004 Ishiyama et al. [16] compiled a tree of UDP-hexose 4-epimerases using 56 sequences that had been annotated as UDP-Glc-4-epimerases, and divided them into three groups based on phylogeny and *in vitro* biochemical assays: those with preference for UDP-Glc (group 1), those with an equivalent activity on both UDP-Glc and UDP-GlcNAc (group 2), and those with a preference for UDP-GlcNAc (group 3) [16]. However, those studies predated the experiments discussed above and we undertook a reappraisal of the relationships.

## Materials and Methods

### *Selection of Protien sequences and analysis*

**Figure 1:**
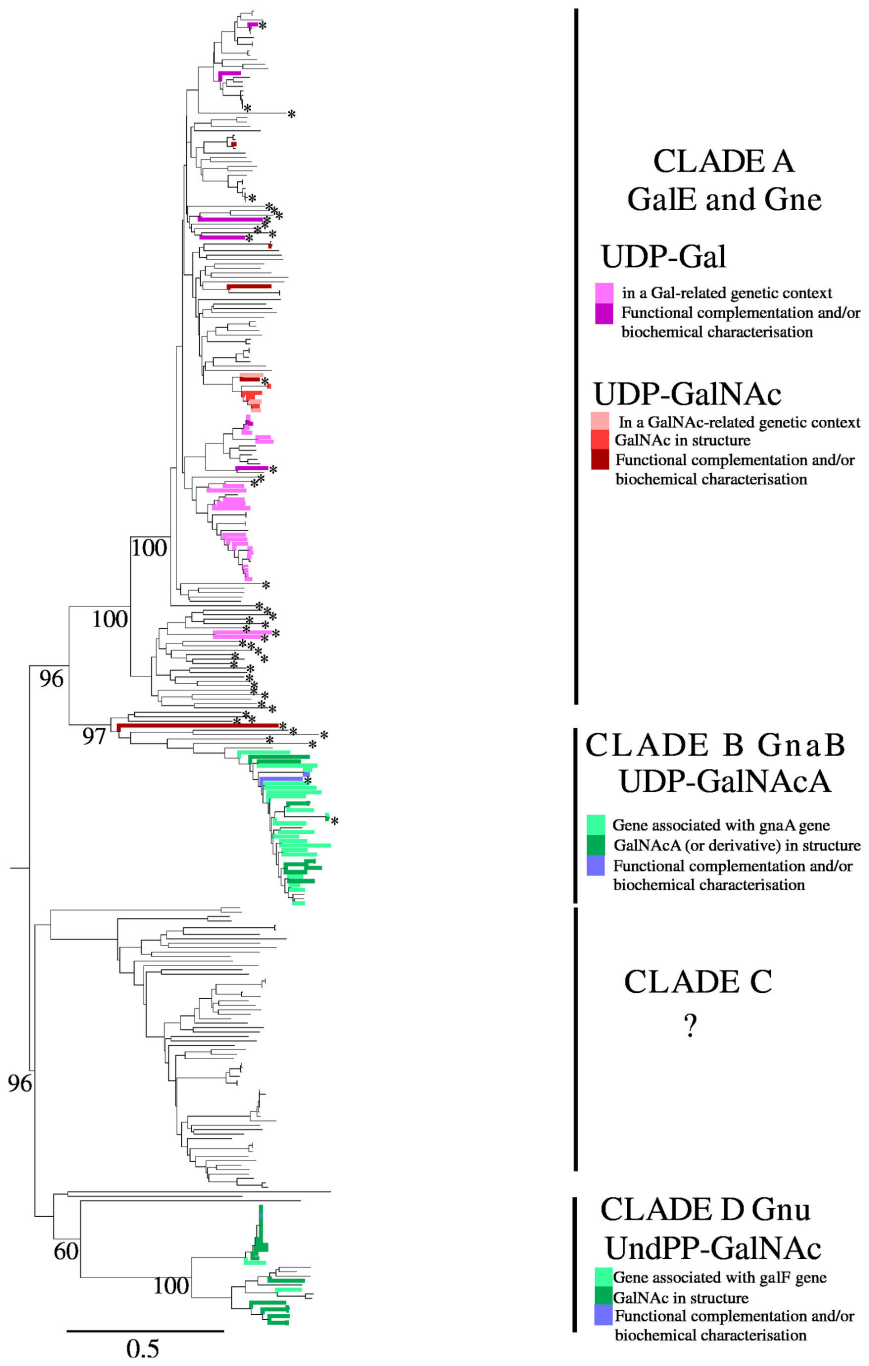
Tree of Gne and related proteins in the*Gammaproteobacteriaceae* and other selected species as found by genome BLAST or taken from the literature as described in the text and listed in Table S1. The tree is based on that shown in Figure S1 in which all of the strains are named. The four major clades are shown with branches coloured where additional information is available as indicated to the right of the Figure and in the text. The proteins indicated by an asterisk were from the Ishiyama et al (16) study.

We assembled a set of protein sequences for analysis using BLASTP with the amino-acid sequences of 1/ Gne2 from *E. coli* O86, 2/ the Gne protein from *E. coli* O157, 3/ WbpP from *P. aeruginosa* O6, and 4/ Gne from *Y. enterocolitica* O8, that represent the diversity of UDP-GlcNAc and UndPP-GlcNAc 4-epimerases that have been characterised in recent years. These four proteins belong to the NAD-dependent epimerase/dehydratase family, PF01370. We searched only genome sequences and confined the BLASTP search to the *Gammaproteobacteria* to keep the output to a manageable level. Additional Gne protein sequences were extracted from known OPS gene cluster sequences of several species of *Enterobacteriaceae*, and the original 56 sequences from the Ishiyama study [16] were also included (Table S1). These 56 proteins added taxonomic depth to this study by including several firmicute, archae and eucaryote proteins as well as the characterised human, *Bacillus* *subtilus* and *E. coli* GalE proteins. After excluding duplicates there were 295 proteins that were aligned using the CLUSTAL program, and the alignment was used to construct a neighbour-joining tree by MEGA (Figure 1 Figure S1). Bootstrap values above 50% are shown in Figure S1 adjacent to nodes (percentage of 1000 bootstrap trees that contain the node), and values for major nodes are shown in Figure 1.

### *Distribution of gnu and gne genes in E. coli serotype* strains

We took a set of *E. coli* strains to represent serogroups for which an O antigen structure has been determined, and for which we have the O-antigen gene cluster from sequence data, which is to be published separately. We excluded O antigens with the genes of the ABC transporter pathway and one with an initial sugar transferase gene. The 115 that remained had *wzx* and *wzy* genes in the gene cluster, so are assumed to use GlcNAc or GalNAc as the first sugar. We undertook two PCR reactions on each to test for the presence of a clade D *gnu* gene, using primers either based on sequence of the *gnu* gene of the *E. coli* O157 strain [8] (5-ACAGATTGGTGATGTTCG-3 and 5-ATCAAAGCAATATCCACC-3), or sequences between *gnu* and upstream gene *galF* (one primer in *gnu* (5-ATCGAACTCTGCAGTATGAATT-3), and the other in *galF*(5-ATGTACTGAATCATTGGCTTGT-3)).

## Results and Discussion

It was clear from the recent data on variation in their functions that there is a need to update the nomenclature for proteins that had been identified as Gne epimerases. We undertook an extensive survey of UDP-GlcNAc and UndPP-GlcNAc-4-epimerases and related proteins as described in Materials and Methods, and constructed a neighbour-joining tree as shown in Figure 1. Most of the 295 proteins studied fell into four distinct clades that were named clades A, B, C and D and are discussed below.

### *Clade A* - *galE and gne genes* - *UDP-Glc epimerases and UDP-GlcNAc epimerases*

Clade A epimerases include those that act on UDP-GlcNAc in *E. coli* O86, *Y. enterocolitica* O8, and *Y. pseudotuberculosis* O6 and O7, and are the Rush et al. [8] homology group of epimerases for OPS with GalNAc residues that were not the first sugar in the repeat unit. Clade A proteins also include the original UDP-Glc/UDP-Gal 4-epimerases known as GalE, including the human and *E. coli* forms and others from *Trypanosoma* , *Streptococcus thermophilus*, *B* *. subtilus* , *Pasteurella* *multicoida* , *Erwinia* *amylovora* , and *Avibacterium* *paragallinarum* that are spread throughout this clade. Some of the UDP-Glc/UDP-Gal 4-epimerase functions is supported by their genes being associated with other genes of the *gal* operon. Thus for clade A, there are two known functions.

The clade A Gne proteins are the original UDP-GlcNAc epimerases, and we propose that they retain that name, but those in the other clades are now shown to have different functions and receive new names. In a few cases it has been shown that the substrate specificity of clade A proteins is due to the size of the substrate-binding pocket as discussed in the introduction. In such cases the pocket size determines if UDP-GlcNAc and UDP-GalNAc can enter the site, but discrimination against UDP-Glc or UDP-Gal has not been tested when the active site was made larger by amino acid substitution.

We are not aware of any protein in the other clades being primarily a UDP-Glc or UDP-GlcNAc epimerase although, as discussed above, some are known to have one or both functions *in vitro*. We recommend that the use of “*galE*” and “*gne*” names be confined to those in clade A unless there is experimental evidence to the contrary, with the default name being “*galE*” unless there is reason to use “*gne*”.

### *Clade D -gnu genes* - *UndPP-GlcNAc epimerases*

Clade D proteins include those for the known UndPP-GlcNAc epimerases. Clade D corresponds to the homology group identified by Rush et al. [8] for Gne proteins associated with repeat units having GalNAc as the first sugar. The substrate is quite different to that of the traditional Gne epimerase substrate, UDP-GlcNAc, and in addition, the O157 enzyme, when tested, did not show any activity towards UDP-GlcNAc. We propose the name *gnu* for these genes. There is support for many of these having the UndPP-GlcNAc epimerase function. For the 15 proteins from *E. coli* (including *Shigella* ), *S. enterica*, *Citrobacter* , *Cronobacter* , *Edwardsiella* or *Serratia* , the corresponding gene is located upstream of the OPS gene cluster at the same locus as for the characterised *E. coli* O157 UndPP-GlcNAc epimerase.

There is good statistical support for this gene being associated with GalNAc residues. Wang et al [7] showed by PCR that 14 of 22 *E. coli* with GalNAc in the structure had the clade D gene, but only 4 of the 40 strains without GalNAc were positive. There are now many more available O-antigen structures so we extended this analysis to cover 115 *E. coli* serogroups, and also included information on presence of a *gne* gene in the gene cluster from sequence data to be published separately. The 115 *E. coli* O antigen gene clusters include a *wzx* and *wzy* gene but lack an initial transferase gene. All of them have either or both of GlcNAc and GalNAc residues, and these are assumed to include the first sugar. They are divided into 6 groups based on the patterns of GlcNAc and GalNAc in the structures (Table S2). Each pattern gives a specific expectation in terms of presence of the *gna* and *gne* genes as shown. In group 1 there is a main-chain GalNAc and a side-chain GalNAc but no GlcNAc, so one GalNAc has to be the first sugar and the other is not, so both *gnu* and *gne* are needed. In group 2 there is a main-chain GalNAc and a main-chain GlcNAc. Our O-unit structures do not tell us which sugar is the first, so either *gnu* or *gne* is needed depending on which is the first sugar. The other patterns give different predictions as shown in Table S2, together with our expectations for *gnu* and *gne*. The data is also shown in Table S2. For 101 of the O-antigen structures (groups 3-6) we can determine the needs for *gnu* and *gne* genes independently and for groups 2 and 3 we can infer the need for one or other of the *gnu* and *gne* genes depending on whether a single main-chain GalNAc residue is the first or a later sugar. We find a remarkably close fit for both genes to expectation providing very strong support for the functions proposed for clade B and D genes in *E. coli*. In 104 serogroups the *gnu* gene is present or absent as expected on the basis of chemical structure, and likewise in 105 serogroups for the *gne* gene. There are only 7 cases of a *gnu* or *gne* gene missing when it is expected and only 10 cases where a gene is present when not predicted (Table S2). We propose the name *gnu* for the clade D genes. There are additional proteins from *Shigella* , *S.* enterica, *Citrobacter* , *Cronobacter* , *Edwardsiella* and *Serratia* , for which the corresponding gene is located upstream of the OPS gene cluster at the same locus as for the characterised *E. coli* O157 UndPP-GlcNAc epimerase.

We discussed above that for *Y. pseudotuberculosis* O6 and O7, with both clade A and D proteins, a functional assignment can be made based on the OPS sequence and structure. This clade also includes the *Y. pseudotuberculosis* O3, O10 and O15 Gnu proteins that are close to the O6 and O7 proteins in the tree and 5 additional *Y. pseudotuberculosis* epimerases not included in the tree (O1c, O2b, O2c, O4a, O5a and O5b) [17–20] that are very similar to the others, a total of 11 *Y. pseudotuberculosis* serotypes with a *gnu* gene [17–20], all of which have GalNAc as first sugar. Serotypes O1b, O11 and O12, with GlcNAc as first sugar, have generally similar gene clusters to those of O1c, O2b, O2c, O3, O4a, O5a, O5b and O15, but lack the *gnu* gene (see alignment in [18]). The distribution of *gnu* in *Y. pseudotuberculosis* correlates absolutely with the presence of GalNAc as first sugar, and this again gives very strong support for these members of clade D having Gnu function. We have 35 proteins in clade D if we include all of the *Y. pseudotuberculosis* serotypes listed above, with good support for the *gnu* function for 26 of them.

Clade D is the most divergent of the four clades, and is itself quite diverse in sequence with *E. coli* and *Y. pseudotuberculosis* strains the most divergent with differences in 50% of the amino-acid residues for *Y. pseudotuberculosis* O10 and *E. coli* O157. It is important that the best evidence for Gnu function is for these same species. For *E. coli* we have good biochemical evidence for the Gnu function and statistical evidence for association with presence of GalNAc in the structure, and for *Y. pseudotuberculosis* we have 11 sequences with very strong support for the Gnu function.

### *Clade B* - *gnaB genes* - *UDP-GlcNAcA epimerases*

Clade B proteins, where function is known, are involved in the synthesis of UDP-GalNAcA or its derivatives. The clade B proteins are ALL associated with a homologue of WbpO, with the genes either adjacent or, in *Marinobacter* sp. ELB17, separated by two ORFs. It is normal practice and reasonable to generalise from one well-documented example, and we assume that this pair of proteins generally act in the same way as documented in *P. aeruginosa*. We propose that the clade B epimerase genes be named *gnaB*, with the genes for the oxygenation step becoming *gnaA* (previously *gna*) genes. This proposal fits the standard pattern for nomenclature of OPS genes [21,22] by using *gna* for the UDP-GalNAcA pathway, with *gnaA* and *gnaB* named in function order [21,22]. The biological function proposed for these genes is supported by the presence of a GalNAcA residue (or derivative) in the OPS where the structure is known, including a group of *P. aeruginosa* OPS, several *E. coli* OPS and others in *V. cholerae*, and also the Vi antigen of *S. enterica*.

There is also a *Giardia* enzyme near the boundary of clades A and B, which is a UDP-GlcNAc epimerase that lacks UDP-Glc/UDP-Gal 4-epimerase activity [23]. There are several uncharacterised enzymes in this area, all on long terminal branches.

There are now structures of WbpP [16] from *P. aeruginosa* and WbgU from *Plesiomonas shigelloides* [24]. Both papers report substantial differences at the active site, when compared to previously characterised UDP-GlcNAc epimerases, in agreement with the finding that they have different substrates. However although they are now shown to function primarily as UDP-GlcNAcA epimerases, at the time of publication they were treated as UDP-GlcNAc epimerases [15], and UDP-GlcNAcA was not tested as a substrate or in complex with the enzymes.

**Clade C** - a clade of proteins of unknown function. To our knowledge clade C includes only one protein that has been characterised. WbpV from *P. aeruginosa* O6 is thought to be the final C-4 reductase in formation of UDP-D-QuiNAc from UDP-4-keto-D-QuiNAc [15]. Nucleotide 4-keto-sugar reductases are in the same family as the epimerases that we are discussing, and indeed in GalE the epimerisation has been shown to involve a 4-keto intermediate equivalent to the product of the 4-keto-sugar reductases [25]. It is thus not surprising to find a nucleotide 4-keto-sugar reductase grouping with 4-epimerases and it will be interesting to know the functions of other members of this clade.

### *Relationship of earlier groupings to clades A, B and D*

With regard to earlier classifications, the group 3 “GlcNAc-specific” epimerases of Ishiyama et al [16] are in our clade B, which contains proteins now known to be epimerases for UDP-GlcNAcA [12] with no *in vivo* activity on UDP-GlcNAc, that we have renamed *gnaB*. The Ishiyama group 1 and 2 enzymes fall into clade A, which includes all of those with UDP-Glc and/or UDP-GlcNAc as *in vivo* substrates.

### *Recommendations for Annotation*

We suggest that in annotation of genomes, any gene that falls into clades B or D be named *gnaB* or *gnu* respectively. In both cases there are well-documented cases for assignment of function that make this the best choice for annotation. In the case of clade B well-documented genes are the *P. aeruginosa* O6 gene currently annotated *wbpP* gene [12], and the *S. enterica* Vi antigen gene previously named *viaC* [26] and also *tviC* [27].

In the case of clade D we have the *E. coli* O157 UndPP-GlcNAc epimerase gene [8] that was originally named *gne* by us [7]. The *Yersinia* clade D genes also clearly have *gnu* function.

In the case of Clade A we have well documented *gne* genes from *E coli* O86 [10], *Aeromonas hydrophila* O34 [28] and *Y. enterocolitica* O8 [4], and *galE* genes from *E. coli* [29], *Aeromonas hydrophila* O34 [28], *Erwina* *amylovora* [30], *A* *. paragallinarum* [31], *Pasturella* *multocida* [32], *Y. enterocolitica* O8 [4], *B. subtilis* [5] and Human [33].

However there remains a difficulty in annotation of clade A genes, as GalE and Gne proteins cannot as yet be distinguished from each other by sequence alone, and for genome sequences there is often no information on possible OPS involvement. However many *galE* genes are in an operon for utilisation of exogenous galactose (see Figure 1) and association of a clade A gene sequence with *galK* or *galT* genes is a good indication that it is a *galE* gene with its primary role being galactose utilisation. In contrast the presence of a clade A gene in an OPS gene cluster is a good indication that it is a *gne* gene, particularly if there is a *galE* gene elsewhere in the genome. Comparison with other genomes in the same species group may add further clarification.

While the number of detailed studies is low for clades B and D in particular, this is not unusual in annotation, and the extension of the functions across the respective clades is strongly supported by the data discussed in the clade descriptions. However more data is highly desirable and we hope our analysis will encourage further work in the area and provide a framework for selection of strains for further study. If type strains are needed for comparison, then *Y* *. enterocollitica* O8 (*gne*), *E. coli* O157 (*gnu*) and *P. aeruginosa* O6 (*gnaB/wbpP*) are the best characterized. *E. coli* O86 and *Y. pseudotuberculosis* O6 and O7 have both *gne* and *gnu* and may also be useful in this regard.

## Conclusions

We identify three well-delineated clades of N-acetylhexosamine-4-epimerase proteins, which can be used to identify and name genes in annotation: clade A for UDP-GlcNAc epimerase genes (*gne*), which also includes UDP-Glc epimerase genes (*galE*), clade B for UDP-GlcNAcA epimerase genes (*gnaB*), and clade D for UndPP-GlcNAc epimerase genes (*gnu*) (Figure 1). There is also clade C with closest homologues in clades A, B and D, that may yet be another 4-epimerase clade, but the only member for which we have data is not an epimerase but has a related function. This brings clarity to an area that has become quite confusing, as all of these N-acetylhexosamine-4-epimerases had been thought to act on UDP-GlcNAc, due to misunderstandings about the stage in the respective pathway at which epimerisation occurred.

In particular, the distinction between clade A and the others means that the UDP-GlcNAcA (*gnaB*) and UndPP-GlcNAc (*gnu*) genes are easily distinguished from the *gne* genes with which they were originally confused.

The distinction between *gne* and *gnu* for UDP or UndPP based substrates will also be useful for predicting if a GalNAc residue in an OPS is the first sugar of the repeat unit. This is very important as identifying the first sugar in the structure tells us the order in which the GTs act and also the linkage made by the Wzy polymerase.

The distinction between *gne, gnu* and *gnaB* will be particularly useful in the *Enterobacteriaceae*, in which WecA usually initiates O-unit synthesis by making UndPP-GlcNAc. If there is no *gnu* gene in the gene cluster or at the *galF*-linked locus, then it is very likely that the first sugar is GlcNAc. The converse appears to often apply, but is not an absolute ‘rule’ as the *galF*-linked *gnu* locus can segregate independently of the OPS gene cluster and so *gnu* may be present when not required for synthesis. Secondly the presence of a *gne* gene in the cluster makes it very likely that there is a GalNAc residue in the OPS structure that is added from UDP-GalNAc, and therefore is not the first sugar. Application of these ‘rules’ will often allow one to predict the first sugar when structure and gene cluster are known.

The ability to predict the residue most likely to be first in assembly of the repeat unit will allow new repeat unit structures to be presented as the biological repeat unit. We refer to the fact that many structural studies identify the individual OPS linkages but do not distinguish between linkages that are within the repeat unit from those formed during polymerisation between repeat units. This situation arises when the linkage of the first repeat unit to the LPS core is not determined, but was masked by the many more polymerisation linkages for that sugar.

Finally, although our focus has been on OPS, the ability to distinguish *gnaB, gnu* and to some extent *gne* and *galE* genes, is expected to apply generally. To facilitate comparisons of protein sequences with the proteins used in this study we have included the accession numbers of the 295 proteins in Table S1.

## Supporting Information

Figure S1 Tree of Gne and related proteins used in this study. Tree of Gne and related proteins in the *Gammaproteobacteriaceae* and other selected species as found by genome BLAST or taken from the literature as described in the text. This is the tree used to generate the tree in Figure 1, and includes the protein names that are omitted from Figure 1. The accession numbers of the proteins are given in Table S1. Further details of the proteins can be found in the accession data.

Table S1 Names and accession numbers of the genes used in the project.

Table S2 Structural and Genetic information for 115 *E. coli* serogroups. Representatives of 115 *E. coli* serogroups for which we have both the O-unit structure and a gene-cluster sequence are shown. *gnu_galF* PCR and *gnu-gnu* PCR show the success in PCR reactions with the requisite primers. The *gne* gene column indicates those that have a *gne* gene in the gene cluster. The next four colums give the numbers of main-chain and sidebranch GalNAc and GlcNAc residues in the structures, followed by the expectations for *gnu* or *gne* genes to be present in the gene cluster. The remaining columns show how the gene clusters fit these expectations.
