# Supplementary figures and images for: Biosynthesis of UDP-GlcNAc, UndPP-GlcNAc and UDP-GlcNAcA Involves Three Easily Distinguished 4-Epimerase Enzymes, Gne, Gnu and GnaB
Source: PLoS One. 2013 Jun 14;8(6):e67646. doi: 10.1371/journal.pone.0067646 (PMC3682973; doi:10.1371/journal.pone.0067646)

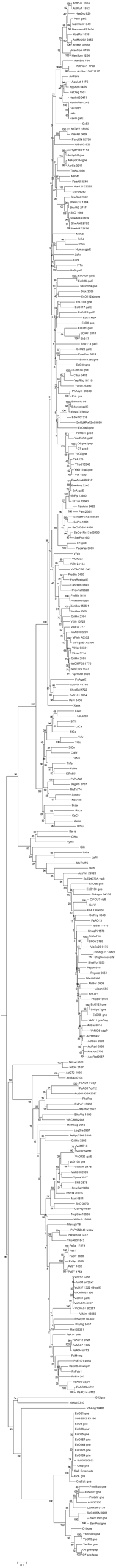

0.1

Supplement: Figure S1 — Tree of Gne and related proteins in the Gammaproteobacteriaceae and other selected species as found by genome BLAST or taken from the literature as described in the text. This is the tree used to generate the tree in Figure 1, and includes the protein names that are omitted from Figure 1. The accession numbers of the proteins are given in Table S1. Further details of the proteins can be found in the accession data. [file pone.0067646.s001.pdf]
