# Supplementary material for: Biosynthesis of UDP-GlcNAc, UndPP-GlcNAc and UDP-GlcNAcA Involves Three Easily Distinguished 4-Epimerase Enzymes, Gne, Gnu and GnaB
Source: PLoS One. 2013 Jun 14;8(6):e67646. doi: 10.1371/journal.pone.0067646 (PMC3682973; doi:10.1371/journal.pone.0067646)
Supplement: Table S1 [file pone.0067646.s002.pdf]

Gene	Accession	Identified by
EcO157_g	NP_288548.1	aa_EcO157gne
ShOn718/318	NP_718745.1	aa_PaO6WbpP
YerEnO8	YP_001007253.1	aa_YeO8
EcO86_gne2	AAO37708.1	aa_EcO86_gne2
Act272_1	ZP_03822729.1	BLAST_EcO157
Act60140	ZP_06785342.1	BLAST_EcO157
ActBau_0_2	YP_002317518.1	BLAST_EcO157
AeHyd796_2	YP_857397.1	BLAST_EcO157
ArN_3033	CBA75633.1	BLAST_EcO157
BegPS_57	ZP_02003541.1	BLAST_EcO157
CanHam_0_1	YP_002923100.1	BLAST_EcO157
Citsp_gn	ZP_04562575.1	BLAST_EcO157
ColPsy_0	YP_267339.1	BLAST_EcO157
CroSak_g	YP_001437279.1	BLAST_EcO157
EcA_gne	ZP_02904249.1	BLAST_EcO157
EcO55_gn	AAL67550.1	BLAST_EcO157
EcO81_gn	YP_002398312.1	BLAST_EcO157
EdwsIct__1	YP_002932741.1	BLAST_EcO157
GriHol_3	ZP_06054119.1	BLAST_EcO157
LegDra_0	ZP_05109139.1	BLAST_EcO157
MarAqVT8	YP_959875.1	BLAST_EcO157
Mari_081	YP_001339680.1	BLAST_EcO157
Mari_083_2	ZP_01738915.1	BLAST_EcO157
MethCap_	YP_113128.1	BLAST_EcO157
MeThio_2	ZP_05105780.1	BLAST_EcO157
NepCae_1	ZP_01167331.1	BLAST_EcO157
NitHal_3_1	YP_003528740.1	BLAST_EcO157
NitHal_3_2	YP_003529031.1	BLAST_EcO157
NitMob_1	ZP_01126033.1	BLAST_EcO157
NitOc_21	YP_344157.1	BLAST_EcO157
PecWas_3	YP_003260419.1	BLAST_EcO157
PhAsym_0_2	YP_003043168.1	BLAST_EcO157
Pho34_20	ZP_01159828.1	BLAST_EcO157
PhoPro	YP_130879.1	BLAST_EcO157
ProtMir_	ZP_03839172.1	BLAST_EcO157
ProvRust_1	ZP_05974444.1	BLAST_EcO157
PsA14_or	YP_790030.1	BLAST_EcO157
PsAO11_w	AAB39483.1	BLAST_EcO157
PsAO12_o	AAM27605.1	BLAST_EcO157
PsAO13_o	AAM27619.1	BLAST_EcO157
PsAO14_o	AAM27632.1	BLAST_EcO157
PsAO17_o	AAM27681.1	BLAST_EcO157
PsAO4_or	AAM27789.1	BLAST_EcO157
PsAO6_wb	AAF23991.1	BLAST_EcO157
PsAPA7_1	YP_001347365.1	BLAST_EcO157
PsEntL48	YP_607182.1	BLAST_EcO157
PsFl_430	YP_261399.1	BLAST_EcO157
PsFl101__2	YP_349782.1	BLAST_EcO157
PsMymp	YP_001187369.1	BLAST_EcO157
PsPgb1	YP_001667620.1	BLAST_EcO157
PsPKT244	NP_743958.1	BLAST_EcO157

PsPuF1_3	YP_001269246.1	BLAST_EcO157
PsPW619_	YP_001748286.1	BLAST_EcO157
PsSa_175	ZP_06459883.1	BLAST_EcO157
PsSP_365	YP_275800.1	BLAST_EcO157
PsST	ZP_05639879.1	BLAST_EcO157
PsST_102	ZP_03396854.1	BLAST_EcO157
PsST_175	NP_791579.1	BLAST_EcO157
PsSyr_36	YP_236708.1	BLAST_EcO157
PsyIng_3	YP_944739.1	BLAST_EcO157
SaE_Gree	AAV34516.1	BLAST_EcO157
SeOdDSM__1	ZP_06639932.1	BLAST_EcO157
SerrOdor	ZP_06190602.1	BLAST_EcO157
SerrProt	YP_001479125.1	BLAST_EcO157
ShB_2878	YP_001051230.1	BLAST_EcO157
ShO_3173	NP_718729.1	BLAST_EcO157
ShwBal_1	YP_002357414.1	BLAST_EcO157
ShwVio_1	YP_003556239.1	BLAST_EcO157
ThioK90_	YP_003460780.1	BLAST_EcO157
VcMO10	ZP_05240744.1	BLAST_EcO157
VcO1_orf	CAA69124.1	BLAST_EcO157
VcO108_g	ADF80967.1	BLAST_EcO157
VcO139_g	Q56623.1	BLAST_EcO157
VcO22_wb	BAA33640.1	BLAST_EcO157
VcO31_ga	ABI85347.1	BLAST_EcO157
VcO37_13	AAM22609.1	BLAST_EcO157
VcV52_02	ZP_01680252.1	BLAST_EcO157
VibAng_1	ZP_01235954.1	BLAST_EcO157
VibMim_3	ZP_06040326.1	BLAST_EcO157
ViChA51_	ZP_01956122.1	BLAST_EcO157
ViChA55_	ZP_01950879.1	BLAST_EcO157
ViChTM21	ZP_04402224.1	BLAST_EcO157
ViMim_06	ZP_05715650.1	BLAST_EcO157
ViMit_00_2	ZP_05883342.1	BLAST_EcO157
ViRC886_	ZP_06081542.1	BLAST_EcO157
Vpara_39	ZP_05118535.1	BLAST_EcO157
VvCMCP6_	NP_760269.1	BLAST_EcO157
YerBer_g	ZP_04630098.1	BLAST_EcO157
YerPsO3_	YP_001721901.1	BLAST_EcO157
YpO15_gn	CAX18366.1	BLAST_EcO157
ActMin_0	ZP_04754582.1	BLAST_EcO86
ActPlu7_	YP_001969146.1	BLAST_EcO86
AerMc	YP_002127352.1	BLAST_EcO86
AggAph_0	YP_003006957.1	BLAST_EcO86
AviPara	ABI18383.1	BLAST_EcO86
Dick_339	YP_003005689.1	BLAST_EcO86
ECIAI1_2	YP_002387517.1	BLAST_EcO86
EcK4_kfo	BAC00525.1	BLAST_EcO86
EcO127_g_1	YP_002329694.1	BLAST_EcO86
EcO22_ga	ABI34566.1	BLAST_EcO86
EcO81_ga	YP_002398310.1	BLAST_EcO86
EcO86_gne1	ZP_05436133.1	BLAST_EcO86
EdwarIcl	AAL25635.1	BLAST_EcO86

EdwsIct__2	YP_002932743.1	BLAST_EcO86
EdwT_013	ZP_06714048.1	BLAST_EcO86
ErA_galE	P35673.1	BLAST_EcO86
ErTas_13	YP_001907273.1	BLAST_EcO86
ErwAmy_2	YP_003531598.1	BLAST_EcO86
GriHol_2_2	ZP_06053383.1	BLAST_EcO86
HaeInPit	YP_002475605.1	BLAST_EcO86
HeaIn86_	YP_248068.1	BLAST_EcO86
IdiBal_0	ZP_01044328.1	BLAST_EcO86
ManSuc_7	YP_087990.1	BLAST_EcO86
Mar121_0	ZP_01075929.1	BLAST_EcO86
Mor_0625	ZP_01896213.1	BLAST_EcO86
Pant_236	ZP_05729045.1	BLAST_EcO86
PatDag_1	ZP_05920640.1	BLAST_EcO86
PhL_gne	NP_931985.1	BLAST_EcO86
ProStu_0	ZP_02958739.1	BLAST_EcO86
PsaAtl_3	YP_662806.1	BLAST_EcO86
PsFl101__1	YP_349563.1	BLAST_EcO86
Sd1012_0	ACD37084.1	BLAST_EcO86
SeOdDSM__2	ZP_06641214.1	BLAST_EcO86
SePoona_	ABP35570.1	BLAST_EcO86
SePro_11	YP_001477393.1	BLAST_EcO86
SerPro_1	YP_001477833.1	BLAST_EcO86
ShB17	ABI55344.1	BLAST_EcO86
ShePu32_	YP_001182910.1	BLAST_EcO86
ShO_1664	NP_717275.1	BLAST_EcO86
ShwAN3_2	YP_870416.1	BLAST_EcO86
ShwMR4_2	YP_734737.1	BLAST_EcO86
ShwMR7_2	YP_738718.1	BLAST_EcO86
ShwW3_27	YP_964090.1	BLAST_EcO86
TolAu_20	YP_002893241.1	BLAST_EcO86
VFish_A0	YP_206310.1	BLAST_EcO86
ViFi_gal	YP_002157945.1	BLAST_EcO86
ViHar_03	YP_001446506.1	BLAST_EcO86
ViHar_37	ZP_01986707.1	BLAST_EcO86
ViMit_00_1	ZP_05882736.1	BLAST_EcO86
YeA125	CAI39182.1	BLAST_EcO86
YeO3gne	CAA87706.1	BLAST_EcO86
YkO11gsk	ABA86989.1	BLAST_EcO86
AcBau381	YP_001715531.1	BLAST_PaO6WbpP
AceJon27	ZP_06064306.1	BLAST_PaO6WbpP
AceRad26	ZP_06073870.1	BLAST_PaO6WbpP
AcHem451	ZP_06726242.1	BLAST_PaO6WbpP
AciRad_0	ZP_05361827.1	BLAST_PaO6WbpP
ActBau_0_1	YP_002317509.1	BLAST_PaO6WbpP
ActDP1	YP_044889.1	BLAST_PaO6WbpP
Alcan_58	ZP_05041062.1	BLAST_PaO6WbpP
AlcBor_0	YP_692629.1	BLAST_PaO6WbpP
AzoVin_2	YP_002800133.1	BLAST_PaO6WbpP
CiFOU7_v	AAK14182.1	BLAST_PaO6WbpP
ColPsy_3	YP_270311.1	BLAST_PaO6WbpP
EcE24377	YP_001463384.1	BLAST_PaO6WbpP

EcO121_g	AAO39691.1	BLAST_PaO6WbpP
EcO138_g	AAZ85711.1	BLAST_PaO6WbpP
EcO35_gn	ACV67284.1	BLAST_PaO6WbpP
EcO98_gn	ABB04484.1	BLAST_PaO6WbpP
IdiBal_1	ZP_01043194.1	BLAST_PaO6WbpP
Mari_083_1	ZP_01738910.1	BLAST_PaO6WbpP
PhAsym_0_1	YP_003043166.1	BLAST_PaO6WbpP
Pho34_19	ZP_01159815.1	BLAST_PaO6WbpP
PlShigO1	BAA85007.1	BLAST_PaO6WbpP
PsA_O6wb	AAF23998.1	BLAST_PaO6WbpP
PsAO13	AAM27613.1	BLAST_PaO6WbpP
PsyArc_0	YP_263944.1	BLAST_PaO6WbpP
Psychr24	YP_001279153.1	BLAST_PaO6WbpP
Se_Vi	NP_458739.1	BLAST_PaO6WbpP
ShDys7_g	AAR97955.1	BLAST_PaO6WbpP
SheWo_16	YP_001760036.1	BLAST_PaO6WbpP
ShigSonn	AAA84870.1	BLAST_PaO6WbpP
ShwaPi_1	YP_002310946.1	BLAST_PaO6WbpP
VibEx25__1	ZP_04922855.1	BLAST_PaO6WbpP
VvMO6_wb	ABD38621.1	BLAST_PaO6WbpP
YkO11_gn	ABB04471.1	BLAST_PaO6WbpP
ActMin20	ZP_03612038.1	BLAST_YeO8
ActPleu1	ZP_00204476.1	BLAST_YeO8
ActPlJL_	YP_001652314.1	BLAST_YeO8
ActSuc13	YP_001345103.1	BLAST_YeO8
AeHyd796_1	YP_855655.1	BLAST_YeO8
AeHydJ1_	ABF59823.1	BLAST_YeO8
AeHydO34	ABA01568.1	BLAST_YeO8
AerSa_32	YP_001142956.1	BLAST_YeO8
AggAct_1	YP_003255777.1	BLAST_YeO8
AltTW7_1	ZP_01612108.1	BLAST_YeO8
AzoVin_4	YP_002801566.1	BLAST_YeO8
CanHam_0_2	YP_002923107.1	BLAST_YeO8
ChroSal_	YP_573773.1	BLAST_YeO8
CitrYon_	ZP_04560078.1	BLAST_YeO8
Citsp_24	ZP_06355367.1	BLAST_YeO8
EcO103_g	ZP_03043871.1	BLAST_YeO8
EcO117_g	ABE98418.1	BLAST_YeO8
EcO128_g	AAO37702.1	BLAST_YeO8
EdwaTEB1	YP_003295255.1	BLAST_YeO8
EnteCan_	ZP_05968336.1	BLAST_YeO8
ErPy_139	YP_002648414.1	BLAST_YeO8
ErwAmy49	YP_003539236.1	BLAST_YeO8
GriHol_2_1	ZP_06053222.1	BLAST_YeO8
HaeDru_8	NP_873335.1	BLAST_YeO8
HaeI_351	CAA40568.1	BLAST_YeO8
HaeIn_ga	YP_001290118.1	BLAST_YeO8
HaePar_1	YP_718999.1	BLAST_YeO8
HaeSom_0	YP_001784580.1	BLAST_YeO8
HaeSom_1	YP_248068.1	BLAST_YeO8
HaIn	NP_438515	BLAST_YeO8
ManHem_1	ZP_04977877.1	BLAST_YeO8

ManHemA2	ZP_05990559.1	BLAST_YeO8
PaM_galE	Q59678.1	BLAST_YeO8
PanAnn_2	YP_003520788.1	BLAST_YeO8
PhAsym_0_3	YP_003043171.1	BLAST_YeO8
ProMir_1	ZP_03840719.1	BLAST_YeO8
ProMirHI	YP_002151674.1	BLAST_YeO8
ProvRet_	ZP_06127560.1	BLAST_YeO8
ProvRust_2	ZP_05974443.1	BLAST_YeO8
PsaHal_0	YP_339009.1	BLAST_YeO8
PsFl_540	YP_262473.1	BLAST_YeO8
PsycCN_0	ZP_01215303.1	BLAST_YeO8
SbBS512_	ACA24755.1	BLAST_YeO8
SeOd4Rx1_1	ZP_06189361.1	BLAST_YeO8
SeOd4Rx1_2	ZP_06191903.1	BLAST_YeO8
SeOd4Rx1_3	ZP_06192008.1	BLAST_YeO8
SheSed_2	YP_001474667.1	BLAST_YeO8
VibEx25__2	ZP_04922762.1	BLAST_YeO8
VibFur_7	ZP_05876662.1	BLAST_YeO8
ViSh_107	ZP_01868022.1	BLAST_YeO8
ViSh_241	ZP_01867915.1	BLAST_YeO8
VpRIMD_2	NP_798779.1	BLAST_YeO8
VvCMPC6_	NP_760654.1	BLAST_YeO8
XenBov_3_1	YP_003469388.1	BLAST_YeO8
YerBerc_	ZP_04630100.1	BLAST_YeO8
YerInt_2	ZP_04635125.1	BLAST_YeO8
YerRho_1	ZP_04614302.1	BLAST_YeO8
Yfred_15	ZP_04631859.1	BLAST_YeO8
Yrh_1920	ZP_04612555.1	BLAST_YeO8
ActLw	T44844	Ishiyama study
BaHa	NP_244245	Ishiyama study
BaS_galE	NP_391765	Ishiyama study
BrJa	NP_774218	Ishiyama study
BrSu	NP_699730	Ishiyama study
CaCr	NP_421186	Ishiyama study
CeEl	NP_493274	Ishiyama study
ClAc	NP_348950	Ishiyama study
ClPe	NP_561425	Ishiyama study
ClPe561	NP_561202	Ishiyama study
CoEf	NP_738423	Ishiyama study
Ec_galE	AAC73846.1	Ishiyama study
EcO113_g	AAD50491.1	Ishiyama study
FrTu	AAN37762	Ishiyama study
FuNe	NP_602894	Ishiyama study
GiIn	AAO39053	Ishiyama study
HeMo	AAN87410	Ishiyama study
Human_ga	Q14376.2	Ishiyama study
LaCa	O84903	Ishiyama study
LaLa	NP_266367	Ishiyama study
LaLa268	NP_268136	Ishiyama study
LaPl	NP_784866	Ishiyama study
LiMo	NP_466000	Ishiyama study
MeLo	NP_108105	Ishiyama study

MeTh275	NP_275523	Ishiyama study
MeTh774	NP_275774	Ishiyama study
MoCa	AAG09980	Ishiyama study
Nos488	NP_488753	Ishiyama study
OcIh	NP_693003	Ishiyama study
OrSJ	BAC02925	Ishiyama study
PiSa	Q43070	Ishiyama study
PsAgalE	NP_250075	Ishiyama study
PsPu745	NP_745273	Ishiyama study
PyHo	NP_143580	Ishiyama study
RhLe	Q59745	Ishiyama study
StCa	AAF25549	Ishiyama study
StCo	NP_627354	Ishiyama study
StPn	NP_346051	Ishiyama study
StTh	AAL67298	Ishiyama study
Syn441	NP_441271	Ishiyama study
ThTe	NP_623502	Ishiyama study
TrBu	CAD23117	Ishiyama study
TrCr	CAE17296	Ishiyama study
ViCh233	NP_233160	Ishiyama study
ViVu	NP_763011	Ishiyama study
XaAx	NP_644047.1	Ishiyama study
EcO104_g	YP_002403332.1	selected gne/galE
EcO112ab	ACD37092.1	selected gne/galE
EcO112ac	ACD37019.1	selected gne/galE
EcO127_g_2	YP_002329696.1	selected gne/galE
EcO130_gn	ACD37156.1	selected gne/galE
EcO143_g	ACA24765.1	selected gne/galE
EcO144_g	ZP_02999452.1	selected gne/galE
EcO6_gne	CAD19796.1	selected gne/galE
EcO8_gne	YP_002387523.1	selected gne/galE
YpO10gne	ADX97409.1	selected gne/galE
YpO6_gne1p	AEP25483.1	selected gne/galE
YpO6_gne2p	AEP25485.1	selected gne/galE
YpO7_gne1p	AEP25503.1	selected gne/galE
YpO7_gne2	AEP25505.1	selected gne/galE
