# Supplementary material for: Biosynthesis of UDP-GlcNAc, UndPP-GlcNAc and UDP-GlcNAcA Involves Three Easily Distinguished 4-Epimerase Enzymes, Gne, Gnu and GnaB
Source: PLoS One. 2013 Jun 14;8(6):e67646. doi: 10.1371/journal.pone.0067646 (PMC3682973; doi:10.1371/journal.pone.0067646)
Supplement: Table S2 — Representatives of 115 E. coli serogroups for which we have both the O-unit structure and a gene-cluster sequence are shown. gnu_galF PCR and gnu-gnu PCR show the success in PCR reactions with the requisite primers. The gne gene column indicates those that have a gne gene in the gene cluster. The next four colums give the numbers of main-chain and sidebranch GalNAc and GlcNAc residues in the structures, followed by the expectations for gnu or gne genes to be present in the gene cluster. The remaining columns show how the gene clusters fit these expectations. [file pone.0067646.s003.pdf]

		Serotype, gene and structure information							Gene expectations				gnu fit to expectation					gne fit to expectation			
Gene-expectation group	Serogroup	gnu-galF PCR	gnu-gnu PCR	gne present in O antigen gene cluster	No. of main-chain GalNAc	No. of side-chain GalNAc	No. of main-chain GlcNAc	No. of side-chain GlcNAc	need gnu	need gne	need gnu OR gne		gnu present and correct	gnu ABsent and correct	gnu missing	gnu in execss of need		gne present and correct	gne ABsent and correct	gne missing	gne in execss of need
1	O102	√	√		1	1			Y	Y			1							1	
	O130	√	√	√	1	1			Y	Y			1					1			
2	O6			√	1		1				Y			1				1			
	O23	√		√	1		1	1			Y		?			?		?			?
	O48			√	1		1				Y			1				1			
	O49			√	1		1				Y			1				1			
	O55	√	√		1		1				Y		1						1		
	O65			√	1		1				Y			1				1			
	O101			√	1		1				Y			1				1			
	O113			√	1		1				Y			1				1			
	O143			√	1		1				Y			1				1			
	O160	√	√		1		1				Y		1						1		
	O178				1		1				Y			?	?				?	?	
	O116			√	1		2				Y			1				1			
3	O103			√	2		1			Y	Y			1				1			
4	O5	√	√		1				Y				1						1		
	O21	√	√		1			1	Y				1						1		
	O24	√	√		1				Y				1						1		
	O46	√	√		1				Y				1						1		
	O71	√	√		1				Y				1						1		
	O104	√	√		1				Y				1						1		
	O120	√	√		1				Y				1						1		
	O124	√	√		1				Y				1						1		
	O131	√	√		1				Y				1						1		
	O140	√	√		1				Y				1						1		
	O146	√	√		1				Y				1						1		
	O147				1				Y						1				1		
	O154		√		1			1	Y				1						1		
	O157	√	√		1				Y				1						1		
	O164	√	√		1				Y				1						1		
	O176				1				Y						1				1		
5	O22	√	√	√	2				Y	Y			1					1			
	O37	√	√	√	2				Y	Y			1					1			
	O76	√	√	√	3				Y	Y			1					1			
	O86	√	√	√	2				Y	Y			1					1			
	O87	√	√		3				Y	Y			1							1	
	O90	√	√		2				Y	Y			1							1	
	O107	√	√	√	2		1		Y	Y			1					1			
		√	√	√	2			1	Y	Y			1					1			
	[illegible]			√	2			1	Y	Y					1			1			
	O117	√	√	√	2				Y	Y			1					1			
	O125	√	√	√	2				Y	Y			1					1			
	O127	√	√	√	2				Y	Y			1					1			
	O128	√	√	√	2				Y	Y			1					1			
	O142	√	√	√	3			1	Y	Y			1					1			
	O158		√		2				Y	Y			1							1	
	O166	√	√	√	2				Y	Y			1					1			
	O1						1							1					1		
	O2						1							1					1		
	O4						1							1					1		
	O7						1							1					1		
	O10						1							1					1		
	O14						1							1					1		
	O16						1							1					1		
	O17						1							1					1		
	O18						1	1						1					1		
	O19						1							1					1		
	O25						1							1					1		
	O26	√	√				1									1			1		
	O29						1							1					1		

	O30						1	1						1					1		
	O35			√	0		1							1							1
	O40						1							1					1		
	O44						1							1					1		
	O54						1							1					1		
	O56						1							1					1		
	O58						1							1					1		
	O64	√	√				1									1			1		
	O68						1							1					1		
	O69						1							1					1		
	O73						1							1					1		
	O75						1							1					1		
	O77						1							1					1		
	O82						1							1					1		
	O83						1							1					1		
	O85						1							1					1		
	O88						1							1					1		
	O98			√			1							1							1
	O100						1							1					1		
	O105						1							1					1		
	O108						1							1					1		
6	O110						1							1					1		
	O111						1							1					1		
	O114						1							1					1		
	O118						1							1					1		
	O119						1							1					1		
	O121			√	0		1							1							1
	O123						1							1					1		
	O126						1							1					1		
	O129						1							1					1		
	O136	√	√				1									1			1		
	O138			√	0		1							1							1
	O139						1							1					1		
	O141						1							1					1		
	O144						1							1					1		
	O148						1							1					1		
	O151						1							1					1		
	O163						1							1					1		
	O172						1							1					1		
	O173						1							1					1		
	O180						1							1					1		
	O3						2							1					1		
	O28						2							1					1		
	O41	√					2									1			1		
	O66						2							1					1		
	O74						2							1					1		
	O91						2							1					1		
	O137						2							1					1		
	O149						2							1					1		
	O150						2							1					1		
	O152						2							1					1		
	O153						2							1					1		
	O159		√				2									1			1		
	O167		√				2									1			1		
	O168						2							1					1		
											**Totals**		**33**	**71**	**3**	**6**		**23**	**82**	**4**	**4**

The alternate yellow and green shading in the lefthand columns indicate the blocks of structure gene combinations discussed in the text.

The shading in the columns for fit to expectation isgreen for fits expectation, red for contrary to expectation and yellow for gene present but not needed for synthesis of the structure. For serogroups O23 and O178, "?" indicates uncertainty as we do not know if the single main-chain GalNAc residue is the first sugar requiring gnu or a later sugar requiring gne, as both or neither gene is present respectively. If that were known then one of the 2 genes would fit expectation and the other would be respectively either excess to expectation or missing.
